# Rab35 Targeting to the Plasma Membrane Is Dependent on the C-terminal Polybasic Cluster

**DOI:** 10.1267/ahc.20-00006

**Published:** 2020-07-22

**Authors:** Katsuhisa Kawai, Youhei Egami, Arata Nishigaki, Nobukazu Araki

**Affiliations:** 1 Department of Histology and Cell Biology, School of Medicine, Kagawa University, Miki, Kagawa 761–0793, Japan

**Keywords:** Rab35, plasma membrane, C-terminus, basic clusters, confocal microscopy

## Abstract

Rab35, a member of the Rab GTPase family, has been implicated in various cellular processes including cell motility and membrane trafficking. Although Rab35 is localized to the plasma membrane, Rab proteins that are identified to have high sequence homology with Rab35 exhibit distinct subcellular localization patterns. Comparing the amino acid sequences between Rab35 and its family members revealed a significant variation in an approximate 30-amino acid region of the C-terminus. This suggests that this region determines the subcellular localization of individual Rab proteins. To confirm this hypothesis, we constructed Rab35–Rab10 chimera proteins by exchanging their C-terminal domains with one another. Confocal microscopy of RAW264 cells expressing EGFP-fused Rab35–Rab10 chimeras has indicated that the C-terminal region of Rab35 is critical for its plasma membrane localization. Furthermore, we were able to determine that a basic amino acid cluster exists in the C-terminal region of Rab35 and that Rab35 localization shifts to the Golgi membrane when the number of basic amino acids in this region is reduced. Thus, it is likely that the approximate 30-amino acid C-terminal region containing basic clusters is responsible for Rab35 plasma membrane localization and that its preferential localization depends on the number of basic amino acids.

## I Introduction

Rab proteins, which are small GTPases belonging to the Ras superfamily, function as molecular switches by cycling activated GTP-bound and inactivated GDP-bound forms. These proteins are involved in various cellular phenomena such as cell motility and cell division, primarily by controlling intracellular membrane traffic or membrane fusion. Mammals have more than 60 types of Rab proteins, and each plays a unique role by specifically localizing to intracellular membrane structures [6, 16, 19, 23]. The majority of Rab proteins undergo prenylation (lipid modification) at carboxyl (C)-terminal cysteine residues, which is catalyzed by Rab geranylgeranyl transferase. The prenylated Rab proteins can bind to membranes through hydrophobic C-terminal prenylated cysteine residues. The prenylation of Rab proteins has been found to be critical for their stable association with membranes [13]. In addition, the amino acid sequence adjacent to the C-terminal lipid modification site has been reported to determine the localization of various Rab proteins to the plasma membrane and/or membranous organelles [5].

Rab35, known to be localized to the plasma membrane, has been implicated in the regulation of endocytic membrane recycling, phagocytosis, and actin cytoskeletal organization [4, 8, 9, 11]. It was reported that the basic amino acid clusters at the C-terminus of Rab35 are the ones required for plasma membrane targeting since the replacement of the C-terminal sequence with a polyethyleneglycol linker results in mistargeting [14]. In this study, we further examined the mechanism of Rab35 plasma membrane localization by engineering chimeric mutants of Rab35 and Rab10 and point-mutated Rab35 genes and subsequently transfecting them into RAW264 cells. This study revealed that Rab35 localization to the plasma membrane is determined by its approximate 30-amino acid C-terminal region and that its preferential localization depends on the number of basic amino acids in the C-terminal sequence.

## II Materials and Methods

### Preparation of plasmids

The pEGFP-Rab35 plasmid was prepared as previously described [9]. The full-length cDNA encoding region of human Rab10 was amplified using PCR. The fragment was then cloned into the Xho and BamH1 restriction sites of the pEGFP-C1 vector (Clontech, Palo Alto, CA). Fusion of the EGFP-tag to the N-terminal side of Rab35 was determined not to affect the intracellular localization of Rab35 [12]. DNA fragments of Rab35–Rab10 hybrid chimeras and point-mutated Rab35 (Rab35-2E and Rab35-4E) were synthesized using GenScript Japan Inc. (Tokyo) and cloned into the pEGFP-C1 vector.

### Cell culture and transfection

RAW264 mouse macrophage-like cells were obtained from the Riken Cell Bank (Tsukuba, Japan) and maintained at 37°C and 5% CO_2_ in Dulbecco’s Modified Eagle Medium (DMEM), which was supplemented with 10% heat-inactivated fetal bovine serum, 100 U/ml penicillin, and 100 μg/ml streptomycin. Cells were transfected with the plasmid vectors as described above using the Neon Transfection System (Life Technologies, Carlsbad, CA) according to the manufacture’s protocol. The cells were seeded onto 25-mm circular coverslips in culture dishes containing DMEM.

### Confocal microscopy and immunocytochemistry

For live-cell imaging, the culture medium was replaced with Ringer’s buffer (RB), which consisted of 155 mM NaCl, 5 mM KCl, 2 mM CaCl_2_, 1 mM MgCl_2_, 2 mM Na_2_HPO_4_, 10 mM glucose, 10 mM HEPES pH 7.2, and 0.5 mg/mL bovine serum albumin (BSA). The 25 mm coverslips were placed into an RB-filled chamber on a 37°C thermo-controlled stage (Tokai Hit INU-ONI, Shizuoka, Japan). Live cells were observed using an inverted microscope equipped with a confocal laser scanning unit (LSM700, Zeiss, Oberkochen, Germany). Time-lapse fluorescence and phase-contrast images of cells expressing EGFP-fused proteins were acquired using a Plan-Apochromat 63× (N.A. = 1.4) objective lens every 15 sec using a 10 mW 488-nm laser at 0.5–1.0% power. The presented image data are representative of at least four independent experiments.

For immunofluorescence microscopy, cells on the coverslips were fixed with 4% paraformaldehyde in phosphate-buffered saline (PBS) for 30 min, washed three times with PBS, and permeabilized in PBS containing 0.1% Triton X-100 and 1% BSA for 10 min. Permeabilized cells were then incubated with anti-GM130 mouse monoclonal antibody (1:200, clone 35/GM130, BD Transduction Laboratories), which is followed by Alexa596-conjugated anti-mouse IgG antibody (1:500, Molecular Probes, Eugene, OR). After washing with PBS, coverslips were mounted in PermaFluor (Thermo Fisher), and observations were made using a Zeiss LSM700 confocal microscope.

## III Results and Discussion

**Fig. 1.**
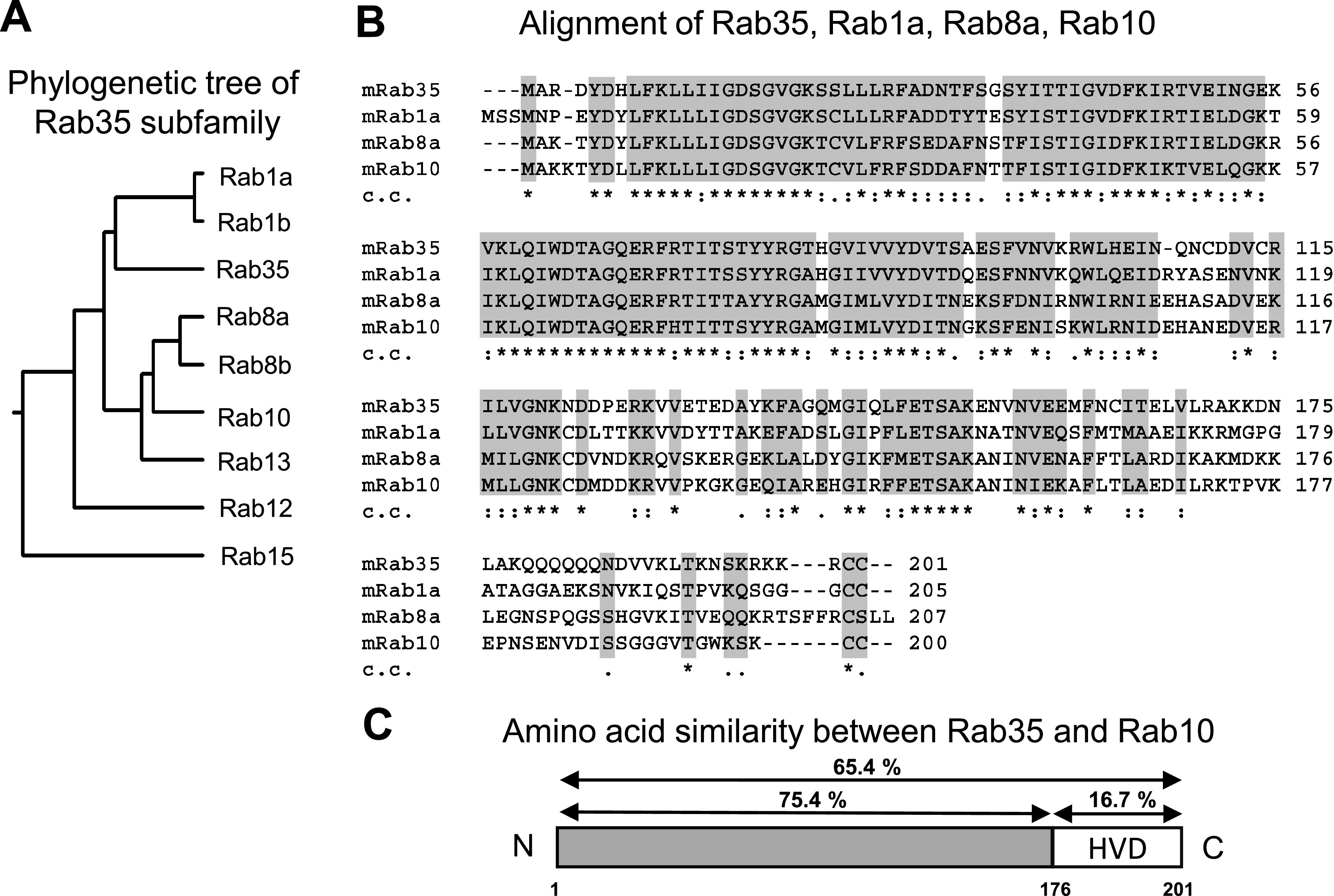
Comparison between the amino acid sequences of Rab35 and its subfamily members. (**A**) Phylogenetic tree of the Rab35 subfamily. The phylogenetic tree was drawn using DNA sequences from the National Center for Biotechnology Information (NCBI) RefSeq database. (**B**) The multiple sequence alignments of mouse Rab35, Rab1a, Rab8a, and Rab10 were created using the Clustal X ver. 2.1 program developed by the European Bioinformatics Institute. In the clustal consensus line (c.c.), an asterisk (*) indicates the positions which have a fully conserved residue, a colon (:) indicates conservation between groups of amino acids with strongly similar properties, and a period (.) indicates conservation between groups of amino acids with weakly similar properties. Conserved and semi-conserved amino acids are shaded light gray. (**C**) Amino acid similarity values between Rab35 and Rab10 were obtained using Clustal X ver. 2.1.

The phylogenetic tree and the multiple sequence alignment of Rab35 subfamily members were generated using the National Center for Biotechnology Information (NCBI) RefSeq database and the Clustal X program (Fig. 1A, B). Comparing the structures of Rab35 and its close relatives revealed that the sequences are highly homologous at the N-terminal side, but are quite variable at the C-terminal end (Fig. 1B). The approximate 30-amino acid C-terminal sequence of the Rab proteins has been identified as the hypervariable C-terminal domain (HVD) [5, 14]. An amino acid analysis of Rab35 and Rab10 revealed that approximately 30 amino acids in their respective HVDs exhibited low similarity (16.7%), while other regions excluding the HVD exhibited 75.4% similarity (Fig. 1C). It is also notable that basic amino acids such as lysine (L) and arginine (R) were found in the HVD of Rab35 (Fig. 1B).

**Fig. 2.**
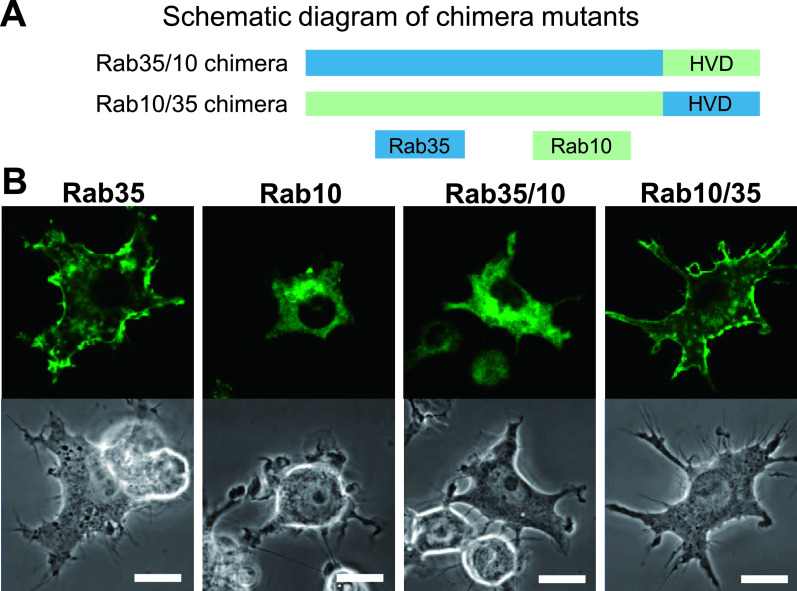
The subcellular localizations of Rab35, Rab10, and Rab35–Rab10 chimeras in live RAW264 cells. (**A**) The schematic diagram showing chimera mutants created by exchanging the C-terminal hypervariable domain (HVD) of Rab35 (blue) and Rab10 (green). (**B**) Confocal microscopy showing the localization of Rab35, Rab10, Rab35/10, and Rab10/35 chimeras in live RAW264 cells. Cells were transfected using a pEGFP-fused Rab protein and observed using a Zeiss LSM700 inverted microscope. An image at one time point from time-lapse microscopy is presented. EGFP-Rab35 was observed on the plasma membrane. EGFP-Rab10 was predominantly observed in the perinuclear Golgi region. EGFP-Rab35/10 chimera exhibited a similar localization to Rab10. EGFP-Rab10/35 chimera was localized to the plasma membrane similar to Rab35. Bars = 10 μm.

For most small G proteins, amino acid sequences at the C-terminus, which are post translationally modified with lipids, are found to be crucial to determine protein localization. With respect to the K-Ras protein, basic clusters at the C-terminus have been shown to play an important role in plasma membrane targeting [20]. It was reported that Rab35 predominantly localizes to the plasma membrane; however, Rab1a and Rab10, which are close relatives of Rab35, do not show plasma membrane localization, but instead localize within the perinuclear Golgi region [7, 18]. Therefore, similar to K-Ras, plasma membrane localization of Rab35 was determined to depend on these basic amino acid clusters. However, it is unclear whether the C-terminal sequence of Rab35 is sufficient for its plasma membrane localization. To examine this issue, we constructed Rab35–Rab10 hybrid chimera mutants by exchanging the C-terminal hypervariable domain (HVD) of Rab35 and Rab10 with one another (Fig. 2A), followed by transfection into RAW264 cells.

By confocal microscopy of live RAW264 cells expressing EGFP-fused Rab35 wild-type, Rab35 with the Rab10 HVD (Rab35/10 chimera), or Rab10 with the Rab35 HVD (Rab10/35 chimera), we have examined the localization of these proteins. Unlike wild-type Rab35, the Rab35/10 chimera was found to be primarily localized to the perinuclear Golgi region, whereas the Rab10/35 chimera exhibited plasma membrane localization similar to that of wild-type Rab35 (Fig. 2B). These findings indicate that the localizations of Rab35 and Rab10 are determined only by their HVD. Therefore, it is likely that the sequence at the C-terminal side of Rab35 is critical for its plasma membrane localization.

**Fig. 3.**
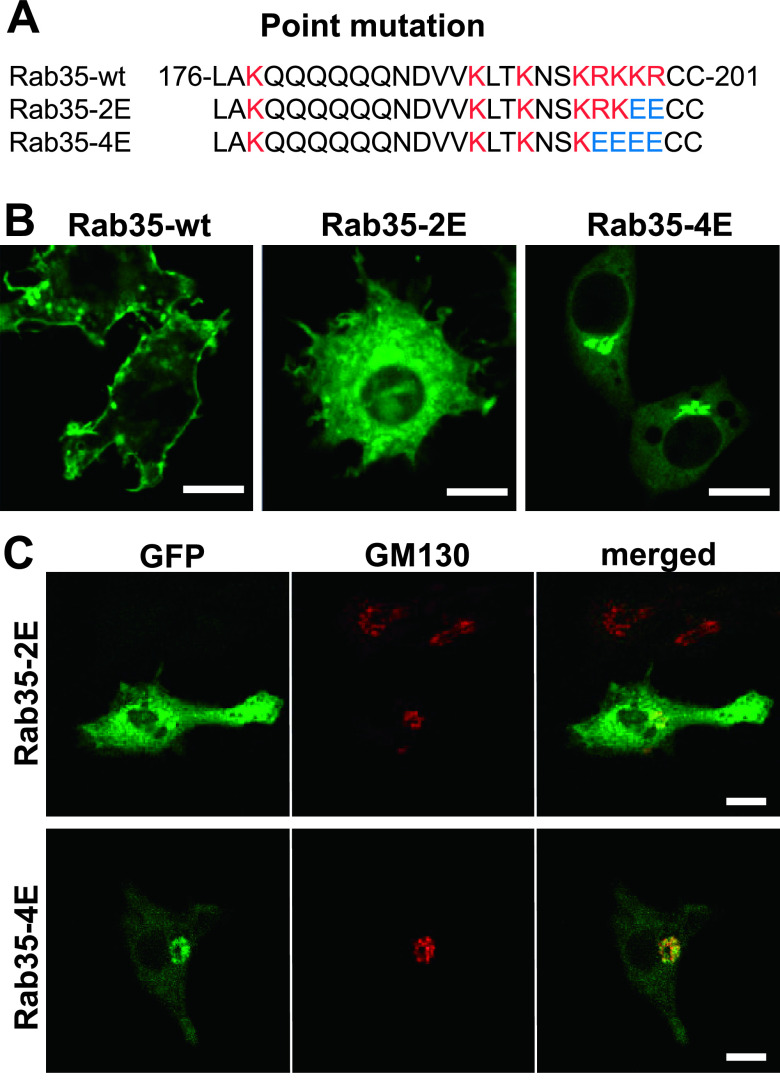
Changes in localization of Rab35 by reducing the number of basic amino acids at the C-terminus. (**A**) The C-terminal amino acid sequences of Rab35-wt, Rab35-2E, and Rab35-4E. The HVD of wild-type Rab35 has a characteristic basic cluster (red letters). In Rab35-2E and -4E, the basic amino acids lysine (K) and arginine (R) were replaced with glutamate (E), an acidic amino acid. The substituted glutamate residues in Rab35-2E and -4E are shown in blue. (**B**) Wild-type EGFP-Rab35, point-mutated EGFP-Rab35-2E, and EGFP-Rab35-4E were transfected into RAW264 cells and observed using a confocal laser microscope. Although wild-type Rab35 was localized to the plasma membrane, Rab35-2E was found to be localized in some organelles in the cytoplasm. Notably, Rab35-4E was exclusively accumulated in the perinuclear region. (**C**) RAW264 cells expressing point-mutated Rab35 (Rab35-2E, Rab-4E) were immunostained with anti-GM130, a *cis*-Golgi marker. By reducing the number of basic amino acids at the C-terminus, Rab35 mutants were accumulated primarily in the Golgi apparatus. Bars = 10 μm.

Next, we have examined how the basic amino acid cluster at the C-terminal side of Rab35 affects its localization. We created two types of point-mutated Rab35 constructs, Rab35-2E and Rab35-4E, by replacing the basic amino acids lysine (K) and arginine (R) in the HVD with the acidic amino acid glutamic acid (E). Rab35-2E and Rab35-4E have two or four exchanged glutamic acid (E) residues in their C-termini, respectively (Fig. 3A). Confocal microscopy revealed that EGFP-Rab35-2E was localized to various intracellular membranes throughout the cytoplasm; however, the plasma membrane did not exhibit intense fluorescence. Notably, EGFP-Rab35-4E was found to be accumulated extensively in the cell center, presumably in the Golgi complex region. The fluorescence of EGFP-Rab35-4E was faintly observed in the plasma membrane and other regions (Fig. 3B). Immunofluorescence cytochemistry for GM130, a *cis*-Golgi marker, revealed that EGFP-Rab35-4E was predominantly localized to the *cis*-Golgi area (Fig. 3C). These results indicated that the localization of Rab35 depends on the number of basic amino acid clusters. Like the results of K-Ras plasma membrane targeting [20], plasma membrane targeting of Rab35 is likely dependent on the electrostatic interaction between the positive charges of the C-terminal basic amino acids and negatively charged lipids in the membrane. Our point mutation experiments of the Rab35 C-terminal residues revealed that the localization of Rab35 shifted from the plasma membrane to intracellular membranous organelles and to the Golgi complex, by reducing the number of basic amino acid residues. As negatively charged acidic lipids such as phosphatidylserine and phosphatidylinositol 4,5-bisphosphate [PtdIns(4,5)P_2_] are more abundantly present in the inner aspect of the plasma membrane, the plasma membrane has a higher affinity for proteins with multiple basic amino acids [22]. In general, intracellular membrane organelles have fewer negative charges compared with the plasma membrane. Among them, the Golgi cisternal membrane was determined to have the least negative charge [1, 2]. This is consistent with our observation that the Rab35-4E mutant predominantly localizes to the Golgi complex, while wild-type Rab 35 localizes to the plasma membrane. Won *et al.* (2006) reported that depletion of PtdIns(4,5)P_2_ and PtdIns(3,4,5)P_3_ dissociates Rab35 from the plasma membrane [21]. Moreover, our previous studies have demonstrated that Rab35 is transiently accumulated in the membrane of forming phagocytic cups, where PtdIns(4,5)P_2_ and PtdIns(3,4,5)P_3_ are abundantly produced [8, 9]. Therefore, it is probable that a cluster of basic amino acids electrostatically interacts with negatively charged membrane lipids such as PtdIns(4,5)P_2_ and PtdIns(3,4,5)P_3_. However, factors other than electric charge may also be involved in the localization of Rab proteins, because there are a variety of membranous compartments to which Rab proteins localize. Rab regulators, including guanine nucleotide exchange factors (GEFs), GTPase activating proteins (GAPs), and guanine nucleotide dissociation inhibitors (GDIs), are deeply involved in determining the intracellular localization of Rab proteins [3, 13, 14, 16, 19]. In fact, the GTP-bound form of Rab35 is localized to the plasma membrane, whereas the GDP-bound Rab35 is diffusely localized in the cytoplasm through binding with a GDI, which shields the hydrophobic geranylgeranyl groups of the C-terminus [10, 15]. Taken together, we propose that, in conjunction with the hydrophobic property of the lipid modification of the C-terminus, the dose-dependent electrostatic interaction of basic amino acids with negatively charged membrane lipids may determine the localization of Rab35.

Some downstream effectors which directly bind to Rab proteins might also affect localization [17]. Although further studies will be necessary to fully understand the complex mechanism by which various Rab proteins are localized, our study was able to provide extensive evidence that the C-terminal electric charge of Rab35 is an important factor for the plasma membrane localization of Rab35 proteins.

## IV Conflicts of Interest

The authors declare that there are no conflicts of interest.

## V Acknowledgments

The authors would like to thank Mr. Kazuhiro Yokoi and Ms. Yukiko Iwabu for their skillful assistance. This study was supported by the Japan Society for the Promotion of Science (grant numbers: 18K06831, 19K07248, 16K08468, 20K07245).

## References

[1] Abe H., Moscarello M. A. and Sturgess J. M. (1976) The distribution of anionic sites on the surface of the Golgi complex. J. Cell Biol. 71; 973–979. 1033190 10.1083/jcb.71.3.973 PMC2109787

[2] Bigay J. and Antonny B. (2012) Curvature, lipid packing, and electrostatics of membrane organelles: defining cellular territories in determining specificity. Dev. Cell 23; 886–895. 23153485 10.1016/j.devcel.2012.10.009

[3] Cabrera M. and Ungermann C. (2013) Guanine nucleotide exchange factors (GEFs) have a critical but not exclusive role in organelle localization of Rab GTPases. J. Biol. Chem. 288; 28704–28712. 23979137 10.1074/jbc.M113.488213 PMC3789967

[4] Chaineau M., Ioannou M. S. and McPherson P. S. (2013) Rab35: GEFs, GAPs and effectors. Traffic 14; 1109–1117. 23905989 10.1111/tra.12096

[5] Chavrier P., Gorvel J.-P., Stelzer E., Simons K., Gruenberg J. and Zerial M. (1991) Hypervariable C-terminal domain of rab proteins acts as a targeting signal. Nature 353; 769–772. 1944536 10.1038/353769a0

[6] Chavrier P., Parton R. G., Hauri H. P., Simons K. and Zerial M. (1990) Localization of low molecular weight GTP binding proteins to exocytic and endocytic compartments. Cell 62; 317–329. 2115402 10.1016/0092-8674(90)90369-p

[7] Chen Y. T., Holcomb C. and Moore H. P. H. (1993) Expression and localization of two low molecular weight GTP-binding proteins, Rab8 and Rab10, by epitope tag. Proc. Natl. Acad. Sci. U S A 90; 6508–6512. 7688123 10.1073/pnas.90.14.6508 PMC46961

[8] Egami Y., Fujii M., Kawai K., Ishikawa Y., Fukuda M. and Araki N. (2015) Activation-inactivation cycling of Rab35 and ARF6 is required for phagocytosis of zymosan in RAW264 macrophages. J. Immunol. Res. 2015; 1–12. 10.1155/2015/429439 PMC4502309 26229970

[9] Egami Y., Fukuda M. and Araki N. (2011) Rab35 regulates phagosome formation through recruitment of ACAP2 in macrophages during FcγR-mediated phagocytosis. J. Cell Sci. 124; 3557–3567. 22045739 10.1242/jcs.083881

[10] Gavriljuk K., Itzen A., Goody R. S., Gerwert K. and Kötting C. (2013) Membrane extraction of Rab proteins by GDP dissociation inhibitor characterized using attenuated total reflection infrared spectroscopy. Proc. Natl. Acad. Sci. U S A 110; 13380–13385. 23898197 10.1073/pnas.1307655110 PMC3746908

[11] Klinkert K. and Echard A. (2016) Rab35 GTPase: A central regulator of phosphoinositides and F-actin in endocytic recycling and beyond. Traffic 17; 1063–1077. 27329675 10.1111/tra.12422

[12] Kouranti I., Sachse M., Arouche N., Goud B. and Echard A. (2006) Rab35 regulates an endocytic recycling pathway essential for the terminal steps of cytokinesis. Curr. Biol. 16; 1719–1725. 16950109 10.1016/j.cub.2006.07.020

[13] Leung K. F., Baron R. and Seabra M. C. (2006) Thematic review series: Lipid posttranslational modifications. Geranylgeranylation of Rab GTPases. J. Lipid Res. 47; 467–475. 16401880 10.1194/jlr.R500017-JLR200

[14] Li F., Yi L., Zhao L., Itzen A., Goody R. S. and Wu Y. W. (2014) The role of the hypervariable C-terminal domain in Rab GTPases membrane targeting. Proc. Natl. Acad. Sci. U S A 111; 2572–2577. 24550285 10.1073/pnas.1313655111 PMC3932868

[15] Müller M. P. and Goody R. S. (2018) Molecular control of Rab activity by GEFs, GAPs and GDI. Small GTPases 9; 5–12. 28055292 10.1080/21541248.2016.1276999 PMC5902221

[16] Pfeffer S. and Aivazian D. (2004) Targeting Rab GTPases to distinct membrane compartments. Nat. Rev. Mol. Cell Biol. 5; 886–896. 15520808 10.1038/nrm1500

[17] Pylypenko O., Hammich H., Yu I.-M. and Houdusse A. (2018) Rab GTPases and their interacting protein partners: Structural insights into Rab functional diversity. Small GTPases 9; 22–48. 28632484 10.1080/21541248.2017.1336191 PMC5902227

[18] Segev N., Mulholland J. and Botstein D. (1988) The yeast GTP-binding YPT1 protein and a mammalian counterpart are associated with the secretion machinery. Cell 52; 915–924. 3127057 10.1016/0092-8674(88)90433-3

[19] Stenmark H. (2009) Rab GTPases as coordinators of vesicle traffic. Nat. Rev. Mol. Cell Biol. 10; 513–525. 19603039 10.1038/nrm2728

[20] Welman A., Burger M. M. and Hagmann J. (2000) Structure and function of the C-terminal hypervariable region of K-Ras4B in plasma membrane targetting and transformation. Oncogene 19; 4582–4591. 11030147 10.1038/sj.onc.1203818

[21] Won D. H., Inoue T., Wei S. P., Man L. K., Byung O. P., Wandless T. J. and Meyer T. (2006) PI(3,4,5)P3 and PI(4,5)P2 lipids target proteins with polybasic clusters to the plasma membrane. Science 314; 1458–1461. 17095657 10.1126/science.1134389 PMC3579512

[22] Yeung T., Gilbert G. E., Shi J., Silvius J., Kapus A. and Grinstein S. (2008) Membrane phosphatidylserine regulates surface charge and protein localization. Science 319; 210–213. 18187657 10.1126/science.1152066

[23] Zerial M. and McBride H. (2001) Rab proteins as membrane organizers. Nat. Rev. Mol. Cell Biol. 2; 107–117. 11252952 10.1038/35052055

